# Development of PET/CT-clinical nomograms for predicting lymph node metastasis in primary lung cancer

**DOI:** 10.1007/s00330-025-12166-z

**Published:** 2025-12-17

**Authors:** Xiaoyu Han, Wentao Li, Julie Lin, Shuoyang Li, Maliazurina B. Saad, Yuliya Kitsel, Simon Heeke, Lingzhi Hong, Mohamed Qayati Mohamed, Xiuning Le, Natalie Vokes, Myrna CB Godoy, Brett W. Carter, Girish S. Shroff, George Eapen, Lauren A. Byers, Ara A. Vaporciyan, Don L. Gibbons, John Heymach, Carol C. Wu, Jianjun Zhang, Jia Wu

**Affiliations:** 1 Department of Imaging Physics, The University of Texas MD Anderson Cancer Center, Houston, USA; 2 Department of Pulmonary Medicine, The University of Texas MD Anderson Cancer Center, Houston, USA; 3 Department of Thoracic Head & Neck Medical Oncology, The University of Texas MD Anderson Cancer Center, Houston, USA; 4 Department of Thoracic Imaging, The University of Texas MD Anderson Cancer Center, Houston, USA; 5 Department of Thoracic and Cardiovascular Surgery, The University of Texas MD Anderson Cancer Center, Houston, USA

**Keywords:** Lung neoplasms, Lymphatic metastasis, Nomograms, Positron emission tomography computed tomography

## Abstract

**Objective:**

Accurate staging of primary lung cancer is crucial for optimizing therapeutic strategies but remains challenging in clinical practice. We aimed to develop a nomogram incorporating clinical characteristics with CT and PET findings to predict lymph node metastasis (LNM) in primary lung cancer.

**Materials and methods:**

We retrospectively analyzed patients with primary lung cancer and mediastinal and hilar LNs from a tertiary care cancer center. All patients underwent endobronchial ultrasound-guided transbronchial needle aspiration, with diagnostic chest CT and PET-CT. Cytological confirmation of transbronchial needle aspiration samples served as the gold standard for diagnosing LNM. We employed an LN-level modeling approach and constructed five models for independent prediction of LNM: (1) Clinical-CT-PET model, (2) Clinical-CT model, (3) PET model, (4) Clinical-PET model, and (5) CT-PET model. Their performance was further evaluated in the subgroup of LNs < 1 cm.

**Results:**

This study included 455 patients (mean age 70 ± 10 years; 55.4% male), predominantly adenocarcinoma (62.0%). Most (68.1%) were stage III–IV. In total, 1391 lymph nodes (1112 training, 279 testing) were analyzed to develop and validate the nomogram. The Clinical-CT-PET model achieved the best diagnostic performance, with AUCs of 0.883 (training cohort) and 0.877 (test cohort), sensitivities of 79.5% and 80.0%, and specificities of 87.1% and 86.9%, respectively. For small LNs, it showed higher AUC (0.797 vs. 0.722, *p* < 0.001) and sensitivity (71.4% vs. 52%) compared to the PET model.

**Conclusion:**

We developed a nomogram that noninvasively estimates the risk of LNM in lung cancer that may inform individualized preoperative assessment and evidence-based decision-making.

**Key Points:**

***Question*** *Can a nomogram integrating clinical, CT, and PET features improve preoperative prediction of lymph node metastasis in primary lung cancer, particularly in small nodes?*

***Findings*** *We developed a Clinical-CT-PET nomogram that achieved the best diagnostic accuracy (AUCs 0.883 and 0.877) among five models, especially for small lymph nodes (< 1 cm).*

***Clinical relevance*** *This noninvasive Clinical-CT-PET nomogram may improve the accuracy of preoperative lymph node staging and guide individualized treatment planning. It may also help avoid unnecessary invasive procedures in lung cancer patients, pending further multi-center validation.*

**Graphical Abstract:**

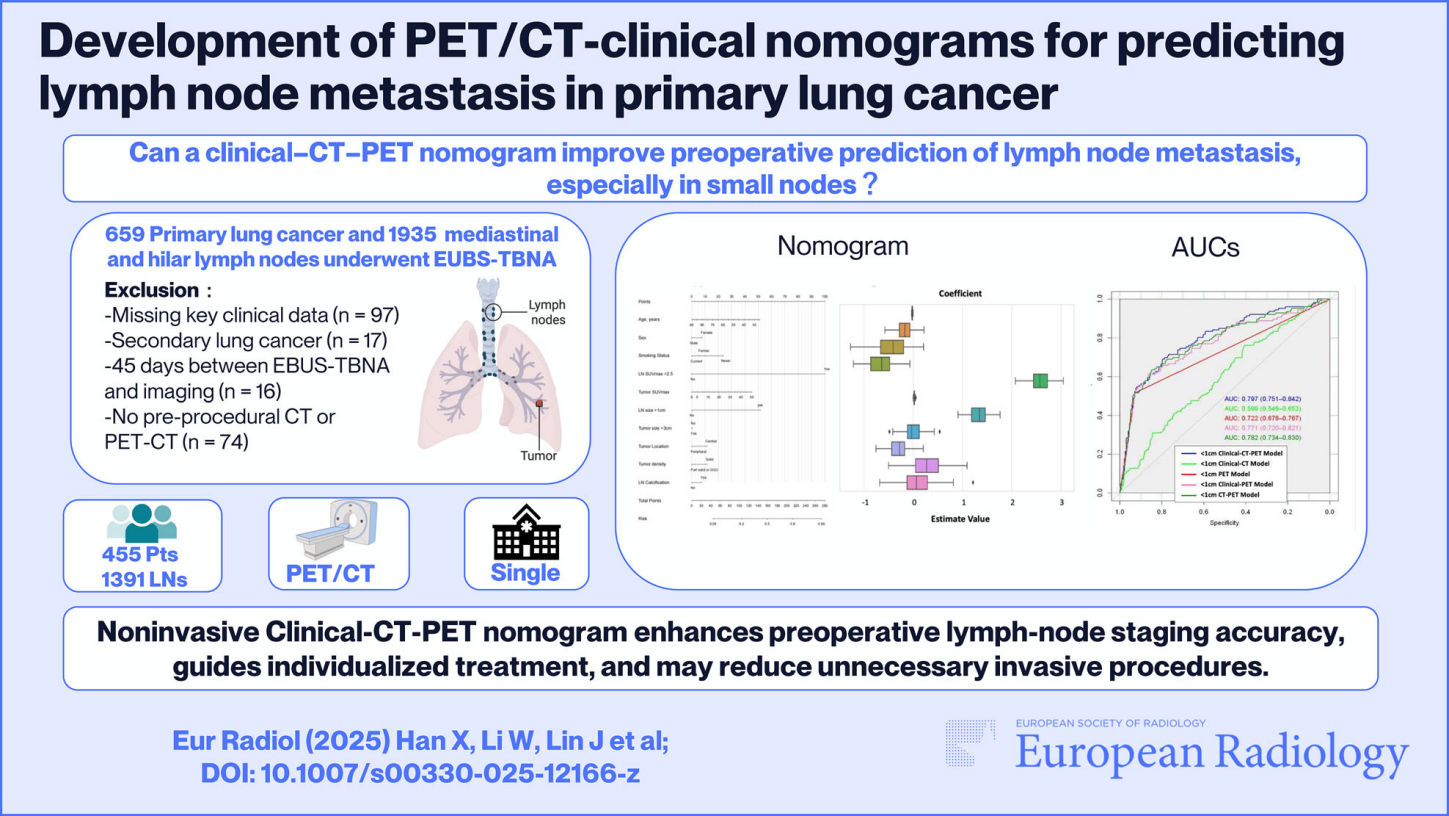

## Introduction

Lung cancer is the leading cause of cancer-related deaths worldwide. Approximately 2.2 million new cases were diagnosed in 2022, making lung cancer the most common cancer globally [1]. Nodal staging, determined by the presence of lymph node metastasis (LNM), helps guide optimal treatment strategies and predict prognosis in patients with lung cancer [2, 3]. For example, N1 disease (stage IIB–IIIA) often requires multimodal management, whereas N2 disease (stage III) is typically treated with neoadjuvant chemoradiotherapy before resection [4]. Thus, accurate staging is essential for tailoring therapeutic interventions, yet it remains challenging in clinical practice. Although mediastinoscopy and endobronchial ultrasound-guided transbronchial needle aspiration (EBUS-TBNA) are the standard procedures for preoperative lymph node staging, access to EBUS remains limited in some regions or institutions. Moreover, even in centers where EBUS is routinely performed, noninvasive imaging-based prediction may help prioritize biopsy targets and improve diagnostic efficiency [5–7].

CT and PET-CT are widely used noninvasive modalities for assessing mediastinal staging in lung cancer. However, CT has shown limited sensitivity (50–60%) and specificity (70–80%) in LN staging [8]. Additionally, the limitations of PET-CT in nodal staging are well documented and arise from various technical, biological, and patient-specific factors [9]. For example, inflammatory or infectious conditions can lead to false-positive results, whereas small metastatic deposits or low metabolic activity may contribute to false-negative results [10].

Nomograms have demonstrated excellent performance in predicting lung cancer risk and prognosis, differentiating benign from malignant lung lesions, and assessing treatment efficacy [11–13]. Previous studies have focused on nomograms for predicting LNM using PET/CT or clinical features [14–16]. However, these studies were limited by small sample sizes, a focus on early-stage cases, single modeling strategies, and a lack of dedicated evaluation for small lymph nodes, which often present diagnostic challenges. Accurate assessment of LNM in advanced-stage lung cancer is particularly critical, as it informs risk stratification, guides therapy selection, and supports personalized treatment planning [17].

Therefore, this study aimed to address these gaps by developing a nomogram that integrates clinical data, CT, and PET findings to noninvasively and preoperatively predict LNM across all clinical stages, including small lymph nodes (< 1 cm), which have been less frequently studied. Key strengths of our study included a large sample size of 455 patients and 1391 lymph nodes, modeling at the lymph-node level, the construction and comparison of five models (Clinical-CT-PET, Clinical-CT, PET, Clinical-PET, and CT-PET) with internal validation, and dedicated validation for small lymph nodes. These features help fill the existing research gap and enhance the clinical applicability of the model.

## Materials and methods

### Ethical approval

The institutional review board approved this study and waived the need for consent (No. 2021-0123). This retrospective cohort study was conducted at a tertiary care cancer center using samples collected from patients from January 2017 through March 2021.

### Study population

**Fig. 1:**
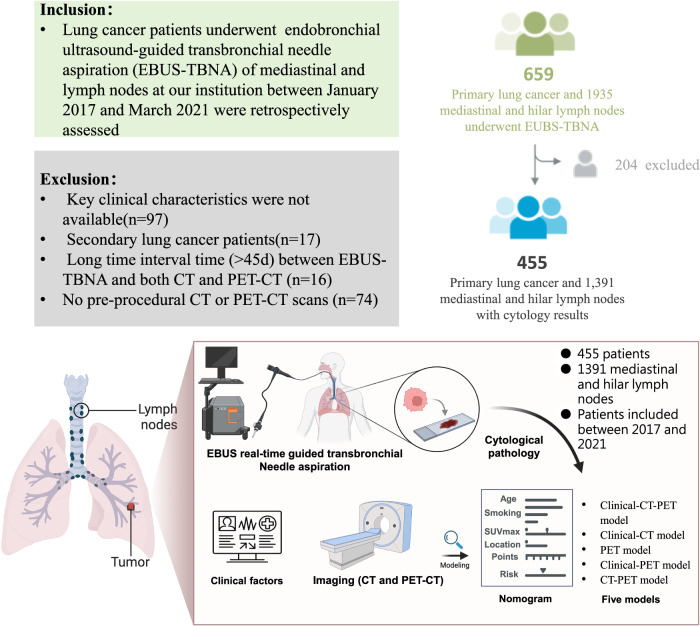
Workflow shows patient selection and exclusion criteria

We searched the database for patients with lung cancer who underwent endobronchial ultrasound-guided transbronchial needle aspiration (EBUS-TBNA) of mediastinal and hilar LNs for staging or diagnostic purposes from January 2017 through March 2021. A total of 659 lung cancer patients with 1935 mediastinal and hilar LNs were retrospectively identified (Fig. 1). Patients were excluded if key clinical characteristics (age, sex, smoking history, and histology) were unavailable, they had secondary lung cancer, more than 45 days gap between EBUS-TBNA and CT or PET-CT, or pre-procedural CT or PET-CT scans were unavailable. Ultimately, 455 patients with 1391 LNs were included.

### Evaluation of clinical and imaging features

Clinical data were retrospectively collected from the electronic medical record (Epic) by a trained research assistant (X.H.) and subsequently verified by thoracic oncologists (J.L., H.L.). Data included demographic characteristics (age and gender), smoking history, underlying chronic obstructive pulmonary disease, history of tuberculosis, TNM stage, and histopathologic classification (adenocarcinoma, squamous cell carcinoma, small cell lung cancer, sarcomatoid carcinoma, adenosquamous cell carcinoma). Smoking history was defined as a lifetime consumption of more than 100 cigarettes, and patients were categorized as current, former, or never-smokers. For current and former smokers, cumulative exposure was quantified in pack-years. TNM staging followed the American Joint Committee of Cancer staging criteria, 4th edition [18]. Tumor histopathologic classification was determined according to the 2021 WHO guidelines.

Two in-house thoracic radiologists (X.H. and K.Y., with 5 and 7 years of experience, respectively) independently reviewed the imaging data. All image assessments were performed in a blinded manner, without access to clinical or histopathologic information. CT data were derived from PET/CT scans in 225 patients and from stand-alone diagnostic chest CT scans in 230 patients. For each patient, LN characteristics—including short-axis diameter on CT, SUVmax, and the presence of calcification—were evaluated. Tumor-related features included SUVmax, size, attenuation, and location. LN or tumor size was defined as the maximum diameter measured on axial CT images; for part-solid and ground-glass nodules, both solid and ground-glass components were included. Tumor attenuation was categorized as solid, part-solid, or ground-glass opacity (GGO). Tumor location was classified as central (involving the segmental bronchi or more proximal airways) or peripheral (located distal to the segmental bronchi), based on bronchial anatomy.

PET/CT imaging protocols were derived from publicly available datasets and institutional standards. Scans were primarily acquired using GE Discovery PET/CT systems (models ST, STE, RX) with a median CT slice thickness of 2.5–5 mm. All patients fasted for at least 6 h, with blood glucose levels confirmed below 200 mg/dL prior to FDG injection. A dose of approximately 259–740 MBq of 18F-FDG was administered intravenously, and PET acquisition began 60–90 min post-injection. Data were corrected for attenuation, scatter, and random events, and reconstructed using OSEM algorithms. Scans included either non-contrast or contrast-enhanced CT, depending on the dataset and clinical context. The maximum standardized uptake value (SUVmax) of the primary tumor and lymph nodes was measured using a dedicated workstation (MIM Software Inc.).

### EBUS-TBNA and pathology diagnosis

At our institution, EBUS-TBNA is routinely performed for mediastinal staging in patients with suspected or confirmed lung cancer prior to treatment planning, including surgery or radiotherapy. The EBUS-TBNA procedure was conducted by experienced board-certified pulmonologists with a 10 MHz convex probe EBUS (BF-UC180F) [19]. All mediastinal, hilar, and lobar LNs with a short axis ≥ 5 mm identified during the procedure are targeted under direct ultrasound visualization. TBNA is then performed with a 22-gauge needle under ultrasound guidance. Systematic EBUS-TBNA staging proceeds sequentially from the N3 hilar stations to the N3, N2, and finally N1 mediastinal stations. If the pathologist detects malignancy (e.g., N3 disease) during rapid on-site examination, the procedure is terminated. For diagnostic purposes, biopsy targets include suspected LNM based on CT, PET-CT, or clinical indications.

The specimen is then spread onto glass slides, fixed, and air-dried. A cytology technician immediately evaluates the dried slides via rapid onsite evaluation to assess their adequacy for further pathological analysis. Alternatively, cell blocks are prepared and analyzed by experienced lung pathologists. LNM is diagnosed based on the presence of malignant cells in conventional stains and/or immunostains, whereas benignity is defined by the absence of tumor cells within a lymphoid tissue background. Specimens lacking lymphoid tissue are considered inadequate and non-diagnostic. Cytological confirmation of TBNA samples serves as the gold standard for LNM diagnosis.

### Nomogram model development

We employed an LN-level modeling approach. We randomly divided the patients into a training cohort (1112 LNs) and a test cohort (279 LNs) in a 4:1 ratio. All variables underwent univariable logistic analysis; variables showing significant correlation (*p* < 0.05) were included in the multivariable analysis to identify risk factors for LNM. Additionally, demographic variables such as age and sex were routinely included due to their clinical accessibility, and LN calcification and tumor location were incorporated based on established associations with LNM [14, 20]. Histologic type was not included because tumor biopsy results are not consistently available, and our goal was to develop a simple, noninvasive model for LN prediction. The training cohort was used to develop the final logistic regression prediction model (i.e., nomogram).

Five independent models were constructed for predicting LNM: (1) the Clinical-CT-PET model, incorporating high-risk clinical (age, gender, smoking history), CT (tumor size (> 3 cm), tumor location (central), tumor density (solid), LN size (> 1 cm), and LN calcification), and PET features (tumor SUVmax (> 2.5) and LN SUVmax (> 2.5)); (2) the Clinical-CT model, including only clinical and CT features; (3) the PET model, including only PET features (LN SUVmax > 2.5); (4) the Clinical-PET model, combining clinical features with PET parameters; and (5) the CT-PET model, combining CT and PET parameters. In the PET model, only LN SUVmax was included, as it is the most widely used PET parameter in clinical practice and serves as a benchmark.

To further test the performance of our nomogram, we conducted a subgroup analysis in LNs with a short-axis diameter < 1 cm to test our Clinical-CT-PET model and PET model. Model components were analyzed, and its performance was assessed on the test set by evaluating both discrimination using the area under the receiver operating characteristic curve (AUC) to distinguish malignant from benign cases and calibration, to assess the agreement between predicted probabilities and observed outcomes. Decision curve analysis (DCA) was used alongside ROC analysis to assess the clinical utility of our model, as it quantifies net benefit across threshold probabilities and determines whether model-based decisions offer greater value than treat-all or treat-none strategies [21]. Differences between AUCs were compared using the DeLong test, and the corresponding Z statistic and *p*-value were reported.

### Statistical analysis

The study utilized SPSS v. 26.0 (IBM) and R v. 4.3.3 software for data analysis. Continuous variables, which were tested for normal distribution, were presented as mean ± standard deviation, whereas categorical variables were expressed as frequency (percentage). Group comparisons were performed using the independent-samples *t*-test (continuous variables), with a two-sided significance level unless otherwise specified, and chi-squared or Fisher’s exact test (categorical variables). The models were constructed via the rms package. A *p*-value < 0.05 was considered statistically significant.

## Results

### Clinicopathologic and imaging characteristics

This study included 455 patients. The mean age was 70 ± 10 years; 252 patients (55.4%) were male (Table S1). Most patients had adenocarcinoma (62.0%), followed by squamous cell carcinoma (31.9%) and other histologic types (6.2%). TNM stages ranged from I to IV, with 310 patients (68.1%) experiencing III and IV stage. Data from 1391 LNs were available; 1112 nodes were used as the training set to develop the nomogram model, whereas data from 279 LNs were used as the test set for performance evaluation. No significant differences in clinical and imaging features between the training and test cohorts were identified (*p* > 0.05; Table 1).

**Table 1:** Clinical, imaging, and lymph node characteristics

Characteristic	All *N* = 1391	Training cohort *N* = 1112	Test cohort *N* = 279	*p*-value
Sex				0.516
Male	757 (54.4)	610 (54.8)	147 (52.7)	
Female	634 (45.6)	502 (45.1)	132 (47.3)	
Age (years), mean ± SD	70 ± 9.5	70 ± 9.5	71 ± 9.5	0.201
Smoking history				0.956
Current	947 (68)	755 (67.8)	192 (68.8)	
Former	251 (18)	202 (18.1)	49 (17.5)	
Never	193 (13.8)	155 (13.9)	38 (13.6)	
Smoking package years	35 (10, 53.5)	36 (10, 53)	31.5 (12.9, 50.6)	0.561
LN				
LN metastasis	387 (27.8)	307 (27.6)	80 (28.7)	0.710
LN size > 1 cm	426 (30.6)	336 (30.2)	90 (32.3)	0.508
LN SUV_max_ > 2.5	429 (30.8)	340 (30.6)	89 (31.9)	0.669
LN calcification	72 (5.2)	59 (5.3)	13 (4.7)	0.663
Primary tumor				
Tumor location				0.681
Peripheral	953 (68.5)	759 (68.3)	194 (69.5)	
Central	438 (31.5)	353 (31.7)	85 (30.5)	
Tumor size > 3 cm	851 (61.2)	672 (60.4)	179 (64.2)	0.254
Tumor density				0.892
Part solid or GGO	186 (13.4)	148 (13.3)	38 (13.6)	
Solid	1205 (86.6)	964 (86.6)	241 (86.4)	0.604
Tumor SUV_max_ > 2.5	15.0 ± 8.5	15.0 ± 8.6	14.9 ± 8.3	0.905
Histologic type				0.240
Squamous cell carcinoma	417 (30)	325 (29.2)	92 (33)	
Adenocarcinoma	834 (60)	679 (61.1)	155 (55.6)	
Other*	140 (10.1)	108 (9.7)	32 (11.5)	

All values are *n* (%) or mean ± SD

*GGO* ground-glass opacity, *LN* lymph node, *SD* standard deviation, *SUV*_*max*_, maximum standardized uptake value

* Including small cell lung cancer, sarcomatoid carcinoma, adenosquamous cell carcinoma and neuroendocrine carcinoma

Clinical and radiologic characteristics were compared between negative and metastatic LNs in both the training and test cohorts (Table 2). LNM occurred more frequently in younger patients (*p* = 0.013) and those without a smoking history (*p* < 0.001) in the training set. In the training set, adenocarcinoma and other histological types were more common in lymph node metastasis, while squamous cell carcinoma was more frequent in benign lymph nodes. However, no significant difference was found in the test set. In both cohorts, LNM was significantly associated with larger LNs (> 1 cm; *p* < 0.001 in both cohorts), higher LN SUV_max_ (> 2.5; *p* < 0.001 in both cohorts), larger tumor size (> 3 cm, *p* = 0.003 in both cohorts), and the likelihood of the LNM to present as solid lesions (*p* < 0.001 in the training cohort, *p* = 0.023 in the test cohort). Other characteristics, including gender, tumor location (peripheral vs. central), presence of LN calcification, and tumor SUV_max_, showed no significant differences between patients with benign and metastatic LNs across both cohorts.

**Table 2:** Clinical and radiologic characteristics of benign versus metastatic lymph nodes

Characteristic	Training cohort			Test cohort		
	Benign *N* = 805	Metastatic *N* = 307	*p*-value	Benign *N* = 199	Metastatic *N* = 80	*p*-value
Sex			0.745			0.271
Male	444 (55.2)	166 (54.1)		109 (54.8)	38 (47.5)	
Female	361 (44.8)	141 (45.9)		90 (45.2)	42 (52.5)	
Age, years, mean ± SD	70.7 ± 9.1	68.9 ± 10.6	0.005	71.2 ± 9.1	70.7 ± 10.4	0.743
Smoking history						0.531
Current	576 (71.6)	179 (58.3)		135 (67.8)	57 (71.3)	
Former	130 (16.1)	72 (23.5)		34 (17.1)	15 (18.8)	
Never	99 (12.3)	56 (18.2)		30 (15.1)	8 (10)	
Smoking package years	39 (12.5, 56)	30 (5, 44)	< 0.001	30 (11.9, 50)	36.5 (20, 52.5)	0.156
LN						
LN size > 1 cm	128 (15.9)	208 (67.8)	< 0.001	37 (18.6)	53 (66.3)	< 0.001
LN SUV_max_ > 2.5	100 (12.4)	240 (78.2)	< 0.001	25 (12.6)	64 (80.0)	< 0.001
LN calcification	38 (4.7)	21 (6.8)	0.178	10 (5.0)	3 (3.8)	0.886
Primary tumor						
Tumor location			0.281			0.640
Peripheral	248 (30.8)	105 (34.2)		59 (29.7)	26 (32.5)	
Central	557 (69.2)	202 (65.8)		140 (70.4)	54 (67.5)	
Tumor size > 3 cm	473 (58.8)	199 (64.8)	0.065	117 (58.8)	62 (77.5)	0.003
Tumor density			0.003			0.023
Part solid or GGO	122 (15.2)	26 (8.5)		33 (16.6)	5 (6.3)	
Solid	683 (84.8)	281 (91.5)		166 (83.4)	75 (93.8)	
Tumor SUV_max_	14.4 ± 8.3	16.5 ± 8.9	< 0.001	14.8 ± 7.3	15.0 ± 8.7	0.826
Histologic type			0.005			0.786
Squamous cell carcinoma	251 (31.2)	74 (24.1)		68 (34.2)	24 (30)	
Adenocarcinoma	488 (60.6)	191 (62.2)		109 (54.8)	46 (57.5)	
Other*	66 (8.2)	42 (13.7)		22 (11.1)	10 (12.5)	

All values are *n* (%) or mean ± SD

*GGO* ground-glass opacity, *LN* lymph node, *SD* standard deviation, *SUV*_*max*_ maximum standardized uptake value

* Including small cell lung cancer, sarcomatoid carcinoma, adenosquamous cell carcinoma and neuroendocrine carcinoma

### LNM nomogram models

**Fig. 2:**
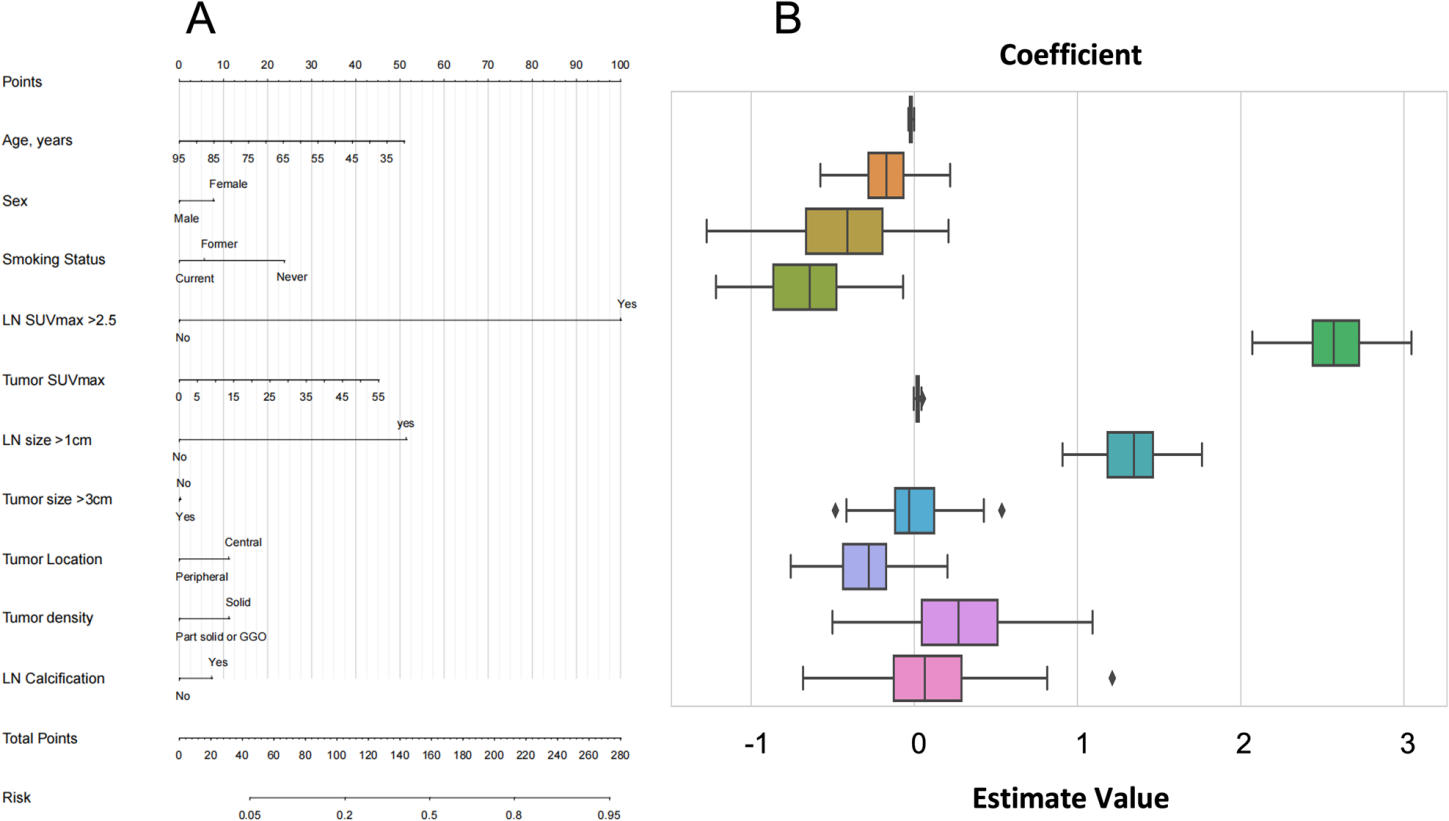
**A** Clinical-CT-PET model nomogram. **B** Coefficient distribution plot. Each box plot corresponds to the variables listed on the left for reference. To use the nomogram, find the position of each variable on the corresponding axis, draw a line to the points axis for the number of points, add the points from all the variables, and draw a line from the total points axis to determine the LNM probabilities at the lower line of the nomogram

**Fig. 3:**
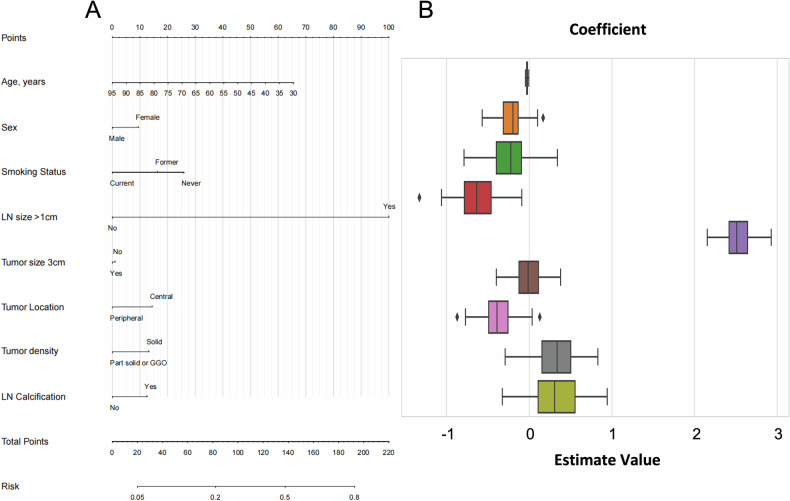
**A** Clinical-CT model nomogram. **B** Coefficient distribution plot. Each box plot corresponds to the variables listed on the left for reference

The Clinical-CT-PET model, which incorporated clinical, CT, and PET characteristics, achieved AUCs of 0.883 and 0.877 in the training and test cohorts, respectively (Fig. 2). Sensitivities and specificities in the Clinical-CT-PET model were 79.5 and 87.1% in the training cohort and 80.0 and 86.9% in the test cohort (Table 3). The Clinical-CT model, incorporating only clinical and CT features, achieved AUCs of 0.801 and 0.763 (Fig. 3), with sensitivities of 79.6% and 66.3% and specificities of 84.0% and 81.4% in the training and test cohorts, respectively. The PET model, incorporating LN SUVmax > 2.5, showed AUCs of 0.796 and 0.825 (Table 3), with sensitivities of 73 and 75% and specificities of 85 and 86.4%. The Clinical-PET model (Fig. S1) achieved AUCs of 0.855 and 0.845, while the CT-PET model demonstrated AUCs of 0.877 and 0.868 (Fig. S2).

**Table 3:** Confounder matrices for different cohorts

Predicted results	Actual results		AUC	Sensitivity (%)	Specificity (%)
	Benign	Metastatic			
Clinical-CT-PET model					
Training cohort			0.883	79.5%	87.1%
Benign	701	63			
Metastatic	104	244			
Test cohort			0.850	80.0%	86.9%
Benign	173	16			
Metastatic	26	64			
LNs < 1 cm cohort			0.797	71.4%	74.9%
Benign	628	36			
Metastasis	211	90			
Clinical-CT model					
Training cohort			0.801	79.6%	84.0%
Benign	676	98			
Metastatic	129	209			
Test cohort			0.763	66.3%	81.4%
Benign	162	27			
Metastatic	37	53			
LNs < 1 cm cohort			0.599	57.1%	53.8%
Benign	451	54			
Metastasis	388	72			
PET model					
Training cohort			0.796	73.0%	85.0%
Benign	684	83			
Metastatic	121	85			
Test cohort			0.825	75.0%	86.4%
Benign	172	20			
Metastatic	27	60			
LNs < 1 cm cohort			0.722	52%	92.9%
Benign	779	61			
Metastasis	60	65			
Clinical-PET model					
Training cohort			0.854	78.2%	87.6%
Benign	705	67			
Metastatic	100	240			
Test cohort			0.845	80.0%	87.4%
Benign	174	16			
Metastatic	25	64			
LNs < 1 cm cohort			0.770	69.1%	71.5%
Benign	600	39			
Metastasis	239	87			
CT-PET model					
Training cohort			0.876	78.2%	87.5%
Benign	704	67			
Metastatic	101	240			
Test cohort			0.850	80.0%	86.9%
Benign	173	16			
Metastatic	26	64			
LNs < 1 cm cohort			0.782	70.0%	72.4%
Benign	607	38			
Metastasis	232	88			

*AUC* area under the curve

**Fig. 4:**
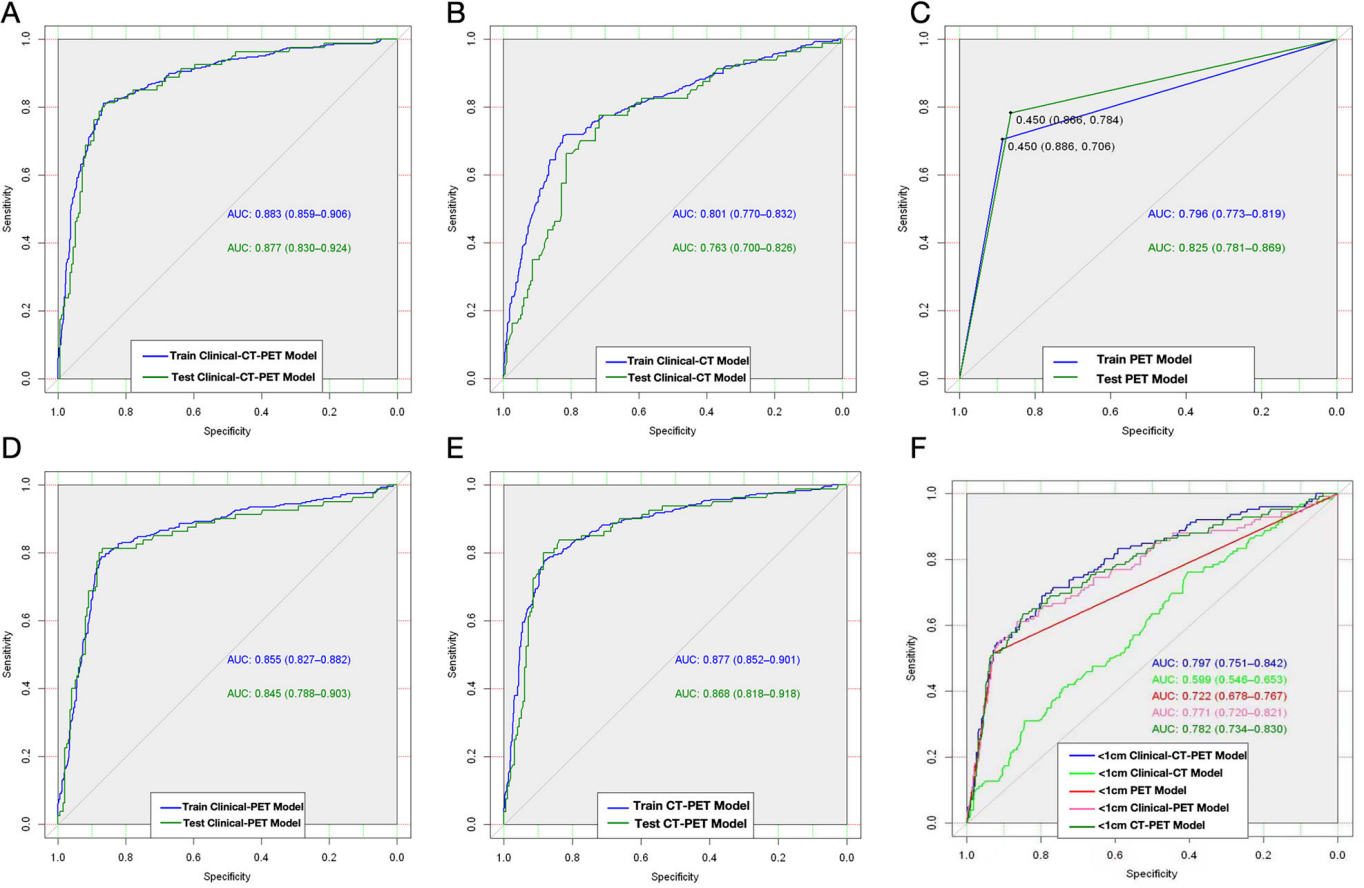
Receiver operating characteristic (ROC) curves of the Clinical-CT-PET model (**A**), Clinical-CT model (**B**), PET model (**C**), Clinical-PET model (**D**) and CT-PET model (**E**) in the training and testing data sets. **F** Subgroup testing of LNs < 1 cm was performed using five models

We compared the performance (AUC) among these five different models (Fig. 4). The AUCs of the Clinical-CT-PET model were statistically significantly higher than those of the Clinical-CT, PET, and Clinical-PET models in the test cohort (*p* < 0.001), but there was no statistically significant difference between the Clinical-CT-PET and CT-PET models (*p* = 0.390).

### Calibration analysis and clinical utility

By examining the calibration curves, we found the predicted probability of LNM to be highly consistent with the actual outcomes, with similar performance observed between the training and test cohorts. For all models, calibration curve analysis showed *p*-values > 0.05 in both the training and test cohorts, indicating good model calibration (Fig. S3). We incorporated DCA to evaluate the clinical utility of our model, the Clinical-CT-PET model demonstrated a higher net benefit than the other four models (Fig. S4).

### Testing nomogram models for small LNs

To evaluate the robustness of our nomogram, we conducted a subgroup analysis restricted to lymph nodes < 1 cm in short-axis, comparing the predictive performance of five models (Table 3, Fig. 4). As shown in Table S2, a total of 965 LNs with a short-axis diameter less than 1 cm were included. Among them, 14.1% LNs showed metastasis, 13.0% had SUVmax > 2.5, and 4.6% exhibited calcification. Most primary tumors were peripherally located (73.4%) and had a solid density (83.9%). In this subgroup, the Clinical-CT-PET model showed a statistically significant higher AUC than the Clinical-CT model (Z = 6.91, *p* < 0.001), the PET model (Z = 2.57, *p* = 0.01), and the Clinical-PET model (Z = 3.54, *p* < 0.001). However, no significant difference was observed when compared to the CT-PET model (Z = 0.93, *p* = 0.35).

## Discussion

In the present study, we constructed a comprehensive nomogram (the Clinical-CT-PT model) capable of noninvasively assessing the risk of LNM across all clinical stages in primary lung cancer. Notably, the model maintained high diagnostic accuracy even for small LNs (< 1 cm), substantially outperforming the corresponding PET model. By providing additional diagnostic insight, our noninvasive model may improve the detection of LNM in primary lung cancers and increase the positive yield of PET and/or CT scans.

Our findings suggested that younger and non-smoking individuals have a higher propensity for LNM. While genomic data were not available, previous studies suggested that more aggressive tumor biology in younger individuals may partly explain this finding [22]. Despite not having traditional risk factors such as smoking, these patients may still present with advanced nodal involvement at diagnosis. It has been well established that lung cancer in never-smokers differs from that in smokers in both molecular characteristics and clinical behavior [23]: Never-smokers are more prone to developing adenocarcinoma and tend to exhibit distinct metastatic patterns, including a greater likelihood of lymphatic spread [24]. These observations highlighted the importance of comprehensive LN assessment, particularly in younger and non-smoking patients. However, the mean age of our cohort was 70 ± 10 years, with only two patients under 40 years old, which limits the applicability of our findings to younger lung cancer patients. While the potential influence of tumor histology on lymph node metastasis was recognized, this variable was intentionally excluded from the model due to the variability in the availability of preoperative histologic confirmation across different institutions. Including histologic type could restrict the model’s generalizability. To improve clinical applicability, our model focused on noninvasive predictors that can be easily obtained from baseline imaging and clinical evaluation.

Compared with traditional CT, PET-CT has been recommended for lung cancer diagnosis and staging due to its superior effectiveness in detecting LNM. We chose SUV_max_ > 2.5 as the cutoff, as it is a widely accepted threshold for differentiating benign from malignant lesions [25]. Our PET model achieved an AUC of 0.796 in the test cohort, with a sensitivity of 73% and specificity of 85%. These findings are consistent with previous literature suggesting that metabolic activity is a valuable marker for nodal malignancy [26]. However, a meta-analysis evaluating fluorodeoxyglucose PET-CT in clinically N0 non–small cell lung cancer patients reported high specificity but relatively low sensitivity for detecting occult nodal metastases. A possible reason for this finding is that many LNM exhibited only mild fluorodeoxyglucose uptake, highlighting the risk of false-negative results when relying solely on SUV_max_ [27]. The superior performance of SUV_max_ observed in our study may be attributed to the predominance of stage III to IV disease in our cohort (68.1%). Advanced-stage lung cancers and LNM often show higher metabolic activity, potentially leading to elevated SUVmax values and better PET-based discrimination in this cohort.

Our Clinical-CT-PET model yielded AUCs of 0.883 and 0.877 in the training and test cohorts, respectively. Furthermore, this model achieved the highest sensitivity and specificity among all models and showed significantly improved diagnostic capability over models based solely on clinical and CT features or LN SUVmax. However, no significant difference was observed when compared to the CT-PET model, suggesting that the inclusion of clinical data may not provide substantial additional benefits in predicting lymph node metastasis. These results align with previous work by Zhang et al [28], who demonstrated that combining imaging features and clinical parameters in a nomogram significantly improves LNM prediction. The superiority of our nomogram suggests that integrating multiple risk factors can better capture the complex biological behavior of LNM. Clinically, such a model may be a valuable tool for individualized preoperative risk stratification and treatment planning. Moreover, our results demonstrated that the Clinical-CT model exhibited similar performance to the PET model in predicting LNM, suggesting its potential as a cost-effective and accessible alternative when PET is unavailable. To further assess model robustness, a subgroup analysis on LNs < 1 cm was conducted, in which the Clinical-CT-PET model still significantly outperformed the PET model, particularly in terms of sensitivity. Inflammatory or fibrotic changes may mimic nodal metastasis on PET, contributing to false-positive predictions [29]. This finding indicated that relying on SUVmax alone was insufficient for evaluating small LNs and that a multimodal approach improves diagnostic accuracy by capturing complementary information.

Our model has a similar performance to previously published models. Pak et al [30] developed a decision-tree model for predicting LNM in non–small cell lung cancer, reporting a sensitivity of 50% and a specificity of 99.28%. Zhang et al [12] constructed a nomogram incorporating tumor size, consolidation-to-tumor ratio, and carcinoembryonic antigen, achieving an AUC of 0.80. Tang et al [14] proposed a predictive model based on Cyfra21-1 and D-dimer levels, tumor size, tumor solidity, and lesion location, with an AUC of 0.904. Our study developed a simplified nomogram that integrated routine clinical and imaging features, which achieved an excellent AUC of 0.883 and balanced specificity and sensitivity. Moreover, while previous studies by Lv et al and Zhu et al [15, 16] developed patient-level nomograms for LNM prediction, our study focused on node-level analysis, offering a more precise and clinically practical approach for preoperative assessment. Hence, our nomogram not only demonstrated robust diagnostic performance but also enhanced clinical applicability through the use of readily available parameters, providing a practical and interpretable tool for preoperative LN-level risk stratification in lung cancer patients.

This study had several limitations. First, this was a single-center retrospective study without external validation, which may limit the generalizability of the model. Differences in imaging acquisition protocols, such as slice thickness, scanner type, and imaging modality, may affect model performance across institutions. Moreover, as access to invasive staging procedures like EBUS-TBNA varies, further validation in multicenter and resource-limited settings is needed. Second, we preselected patients who underwent EBUS-TBNA, which also limits generalizability. In this study cohort, the nodal status is of clinical significance; hence, EBUS-TBNA was performed. For example, in patients with overt active distant metastatic disease, hilar and mediastinal lymph node involvement did not drive treatment decision-making, and hence EBUS-TBNA was unlikely to be performed. Third, radiomics or deep learning methods were not employed for modeling LNM [31, 32]. However, the nomogram approach offers greater interpretability and is more accessible for clinical implementation and routine use of commonly available data. Future work should ideally include direct comparisons between our approach and these advanced methods, particularly on datasets that include higher tumor stages. Lastly, although our nomogram model incorporates multiple predictive factors, it does not account for serum biomarkers or genetic alterations that may further enhance predictive power. Future prospective studies with larger, more diverse populations should integrate molecular or radiomic data to help improve the precision and clinical utility of LNM prediction models.

In conclusion, we developed noninvasive models based on clinical and imaging features to estimate the risk of lymph node metastasis in primary lung cancer. Such a tool could potentially assist clinical decision-making in settings where access to invasive staging methods like EBUS-TBNA is limited. While the model demonstrated promising performance in our internal cohort, further prospective and multicenter studies are needed to assess its generalizability and clinical utility, particularly across diverse care settings.

## Supplementary information

ELECTRONIC SUPPLEMENTARY MATERIAL

## Data Availability

The datasets used and/or analyzed during the current study are available from the corresponding author on reasonable request.

## Abbreviations

AUC: Area under the curve
EBUS-TBNA: Endobronchial ultrasound-guided transbronchial needle aspiration
LN: Lymph node
LNM: Lymph node metastasis
SUV_max_: Maximum standardized uptake value
