# Supplementary material for: Development of PET/CT-clinical nomograms for predicting lymph node metastasis in primary lung cancer
Source: Eur Radiol. 2025 Dec 17;36(5):4110–22. doi: 10.1007/s00330-025-12166-z (PMC13086717; doi:10.1007/s00330-025-12166-z)
Supplement: Supplementary file 1 — ELECTRONIC SUPPLEMENTARY MATERIAL [file 330_2025_12166_MOESM1_ESM.pdf]

Development of PET/CT-Clinical Nomograms for Predicting Lymph Node Metastasis in Primary Lung Cancer

ELECTRONIC SUPPLEMENTARY MATERIAL

Eur Radiol (2025) Han X, Li W, Lin J, et al.

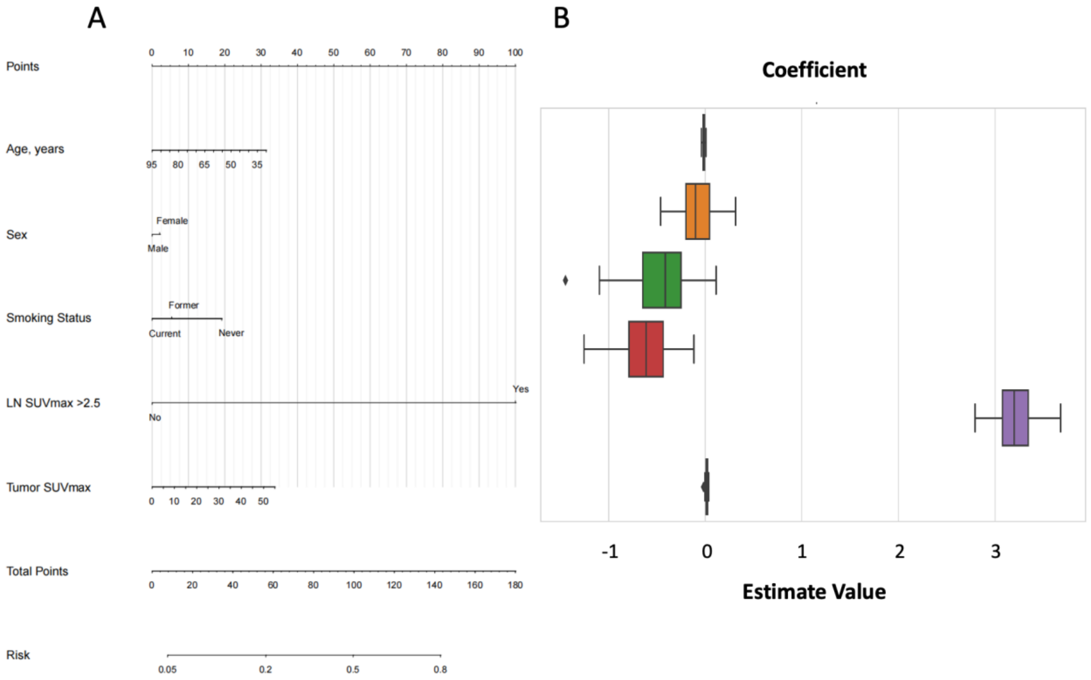

Figure S1 (A) Clinical-PET model nomogram. (B) coefficient distribution plot. Each box plot corresponds to the variables listed on the left for reference.

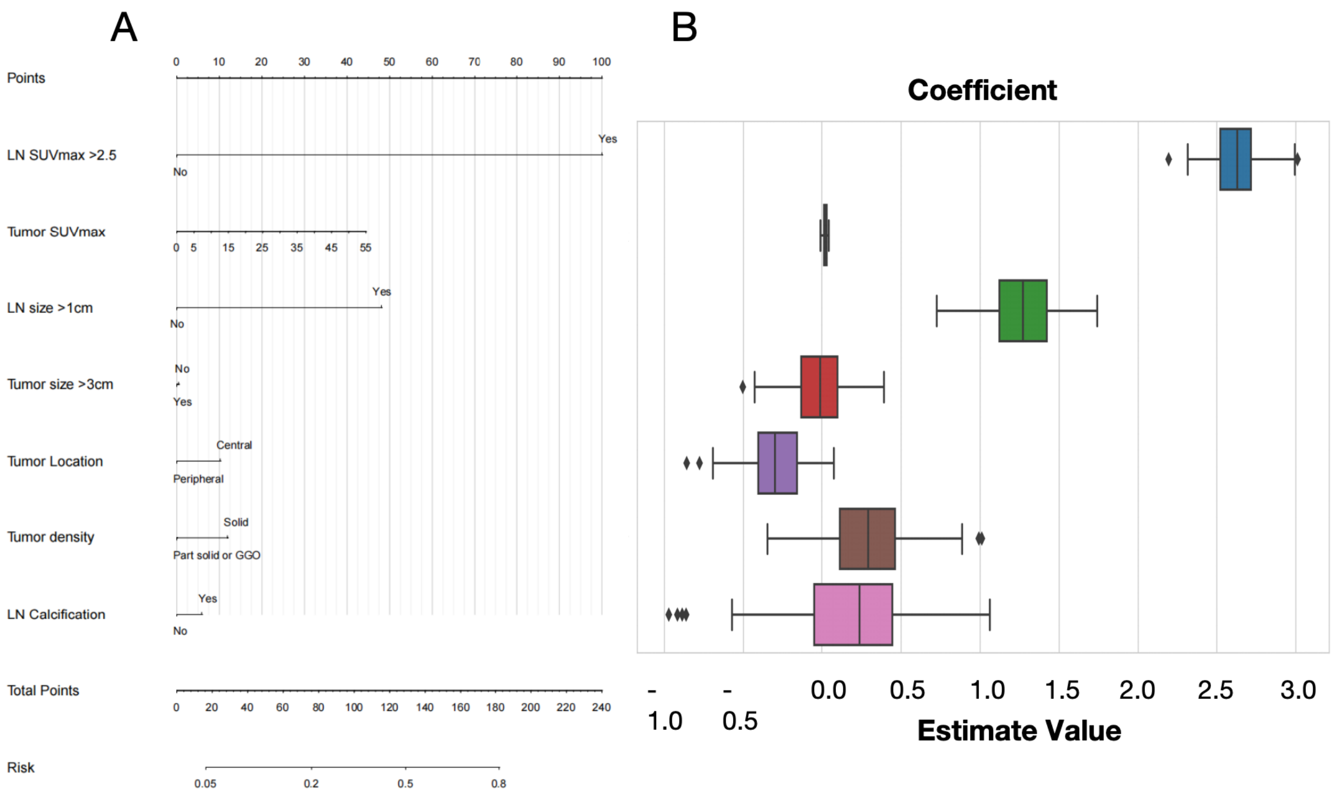

Figure S2 (A) CT-PET model nomogram. (B) coefficient distribution plot.

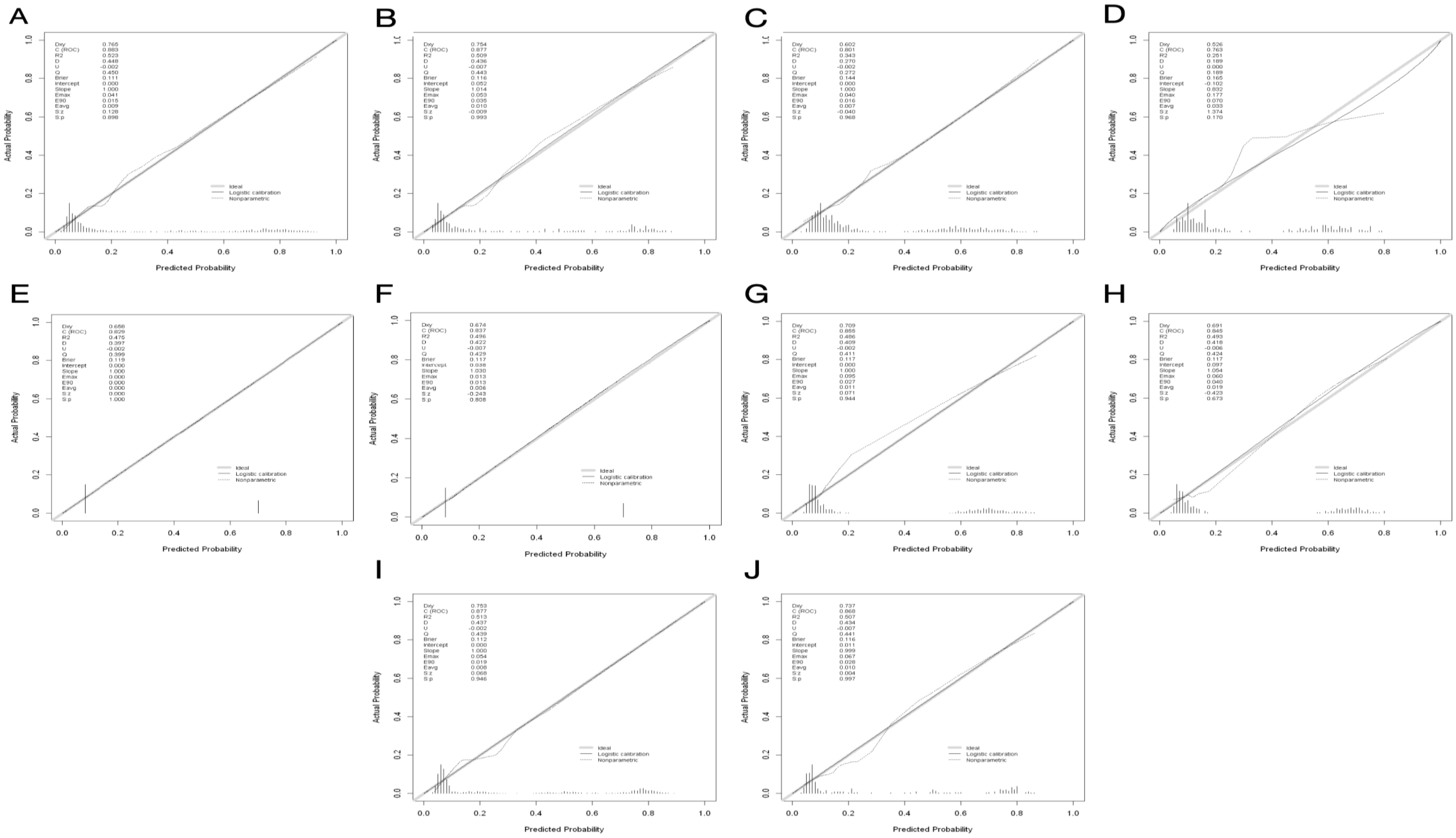

Eur Radiol (2025) Han X, Li W, Lin J, et al.

Figure S3 Calibration curves of the models in the train and test cohorts. (A-B) Clinical-CT-PET model；(C-D) Clinical-CT model; (E-F) PET model.(G-H) Clinical-PET model; .(I-J) CT-PET model.

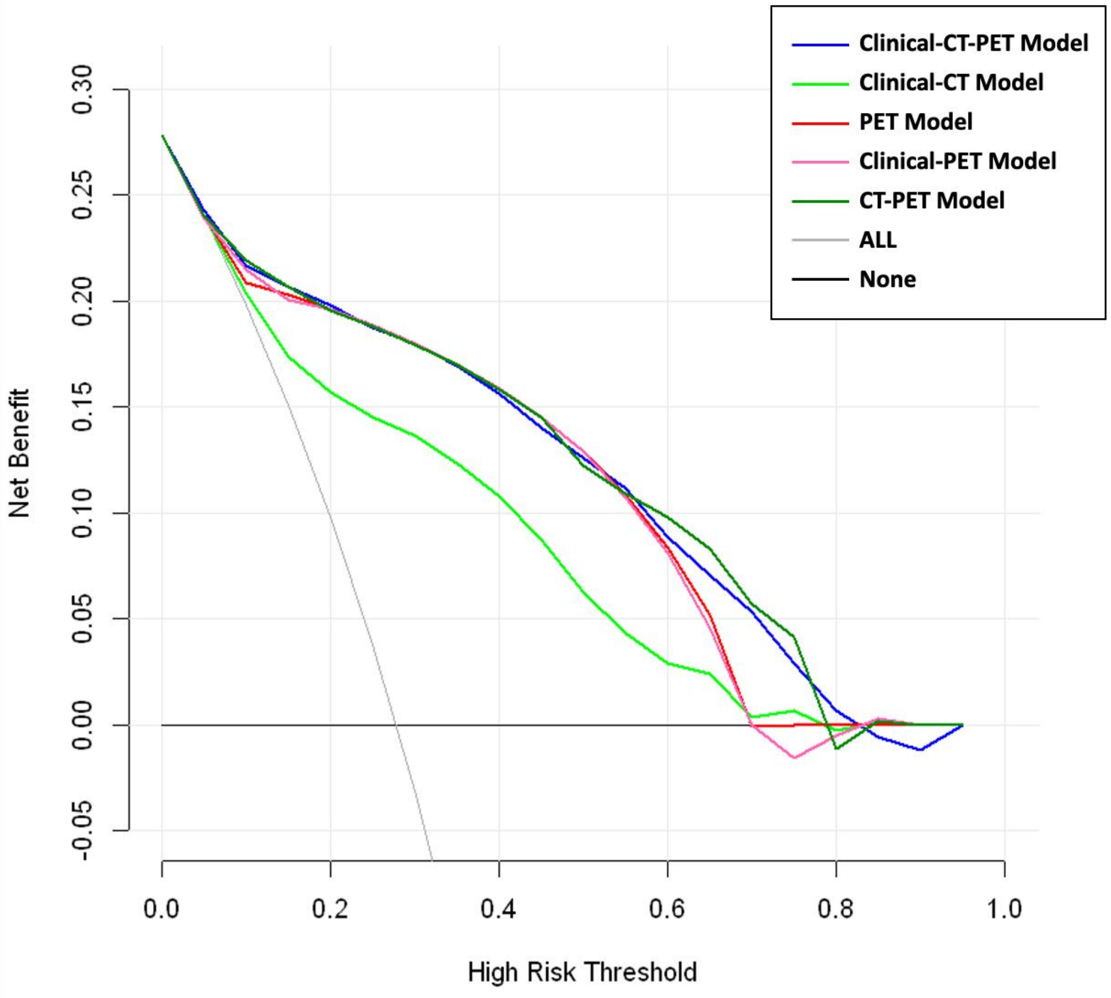

Eur Radiol (2025) Han X, Li W, Lin J, et al.

Figure S4 Decision curve analysis of models predicting lymph node metastasis (LNM) in primary lung cancer patients. The y-axis measures the net benefit. The gray curve represents the strategy of treating all patients while the black horizontal line reflects the treat-none strategy serving as a reference line with zero net benefit.

Table S1. Patient demographic and clinical data.

Characteristic	All patients N=455
Gender	
Male	252 (55.4)
Female	203 (44.6)
Age, years, mean ± SD	70 ± 10
Smoking history (yes)	380 (83.5)
Current	284(62.4)
Fomer	95(20.8)
Never	76(16.7)
Smoking package years	32(7.5,52)
TNM stage	
I	85 (18.7)
II	58 (12.7)
III	199 (43.7)
IV	111 (24.4)
Histologic type	
Squamous cell carcinoma	145 (31.9)
Adenocarcinoma	282 (62.0)
Other*	28 (6.2)
TB history	18 (4.0)
COPD (yes)	106 (23.3)
Tumor size, cm, mean ± SD	4.6 ± 4.3
Tumor density	
Part solid or GGO	53 (11.6)
Solid lesion	400 (87.9)

Tumor SUV_{max} >2.5	340 (74.7)

COPD, chronic obstructive pulmonary disease; GGO, ground-glass opacity; SD, standard deviation; SUV_{max}, maximum standardized uptake value; TB, tuberculosis. * Including small cell lung cancer, sarcomatoid carcinoma, adenosquamous cell carcinoma and neuroendocrine carcinoma.
All values are n (%) or mean±SD.

Table S2. Clinical and imaging data of lymph nodes <1 cm.

Characteristic	All<br>N=965
Gender	
Male	507 (52.5)
Female	458 (47.5)
Age, years, mean ± SD	70 ± 9.5
Smoking history (yes)	815 (84.5)
LN	
LN metastasis	136 (14.1)
LN SUV_{max} >2.5	125 (13.0)
LN calcification	44 (4.6)
Primary tumor	
Tumor location	
Peripheral	708 (73.4)
Central	257 (26.6)
Tumor size >3 cm	558 (57.8)
Tumor density	
Part solid or GGO	155 (16.1)
Solid	810 (83.9)
Tumor SUV_{max} >2.5	777 (80.5)

GGO, ground-glass opacity; LN, lymph node; SD, standard deviation; SUV_{max}, maximum standardized uptake value. All values are n (%) or mean±SD.
